# TET2: a critical regulatory hub with broad therapeutic implications across human diseases

**DOI:** 10.3389/fimmu.2026.1802805

**Published:** 2026-07-31

**Authors:** Jingyi Tang, Ruoxian Wang, Li Long, Wencheng Ma, Xiaofan Han, Hongxue Fu

**Affiliations:** 1 School of Pharmacy and Bioengineering, Chongqing University of Technology, Chongqing, China; 2 Department of Ophthalmology, The First Affiliated Hospital of Chongqing Medical University, Chongqing Key Laboratory for the Prevention and Treatment of Major Blinding Eye Diseases, Chongqing Eye Institute, Chongqing Branch (Municipality Division) of National Clinical Research Center for Ocular Diseases, Chongqing, China; 3 Molecular Biology Laboratory, Chongqing Medical and Pharmaceutical College, Chongqing, China

**Keywords:** TET2, DNA demethylation, epigenetic regulation, cancer, inflammation, metabolic diseases

## Abstract

Ten-Eleven Translocation 2 (TET2) is a pivotal α-ketoglutarate and Fe^2+^-dependent dioxygenase belonging to the TET family, governing epigenetic homeostasis through DNA, RNA, and histone modifications. Its central function involves the iterative oxidation of 5-methylcytosine (5mC) to 5-hydroxymethylcytosine (5hmC) and further derivatives, initiating active DNA demethylation. Beyond this, TET2 catalyzes RNA m^5^C oxidation and interacts with histone modifiers, thus modulating diverse cellular processes. Physiologically, TET2 is indispensable for hematopoietic stem cell (HSC) function, genomic stability, and the resolution of inflammation. Its dysregulation, often driven by somatic mutations, is implicated across a broad spectrum of human diseases. These include hematological malignancies, solid tumors, inflammatory disorders, cardiovascular diseases (CVD), metabolic abnormalities, and neurological conditions. Notably, TET2 exhibits strong context dependency, functioning as a tumor suppressor in myeloid malignancies while exerting distinct immunomodulatory roles in certain solid tumors. This review systematically summarizes recent advances in the molecular mechanisms and physiological functions of TET2, and highlights its disease-specific roles and promising therapeutic strategies. We also discuss unresolved challenges and future research directions to facilitate the clinical translation of TET2-related epigenetic findings.

## 1 Introduction

TET2, a key member of the ten-eleven translocation (TET) family of DNA dioxygenases, orchestrates genomic demethylation, modulates histone modifications, and influences RNA dynamics. As an α-ketoglutarate (α-KG) and Fe^2+^-dependent enzyme, TET2 initiates active DNA demethylation by oxidizing 5-methylcytosine (5mC) to 5-hydroxymethylcytosine (5hmC) (1–3). This crucial step maintains epigenetic equilibrium and can be further extended by subsequent oxidation of 5hmC to 5-formylcytosine (5fC) and 5-carboxylcytosine (5caC), these oxidized cytosine derivatives are recognized and excised by thymine DNA glycosylase (TDG), generating an abasic site that is subsequently repaired through the base excision repair (BER) pathway to restore unmethylated cytosine, thereby completing active DNA demethylation (4–7). In parallel, 5hmC can also be passively diluted during DNA replication in the absence of maintenance methylation (8). Beyond DNA, TET2 directly engages with histone modifiers, regulating gene expression through specific histone marks. At the RNA level, TET2 catalyzes the oxidation of RNA 5-methylcytosine (m^5^C) to 5-hydroxymethylcytosine (hm^5^C), thereby influencing the stability of pluripotency-associated mRNAs during embryonic stem cell (ESC) differentiation and modulating tRNA fragment level (9).

**Figure 1:**
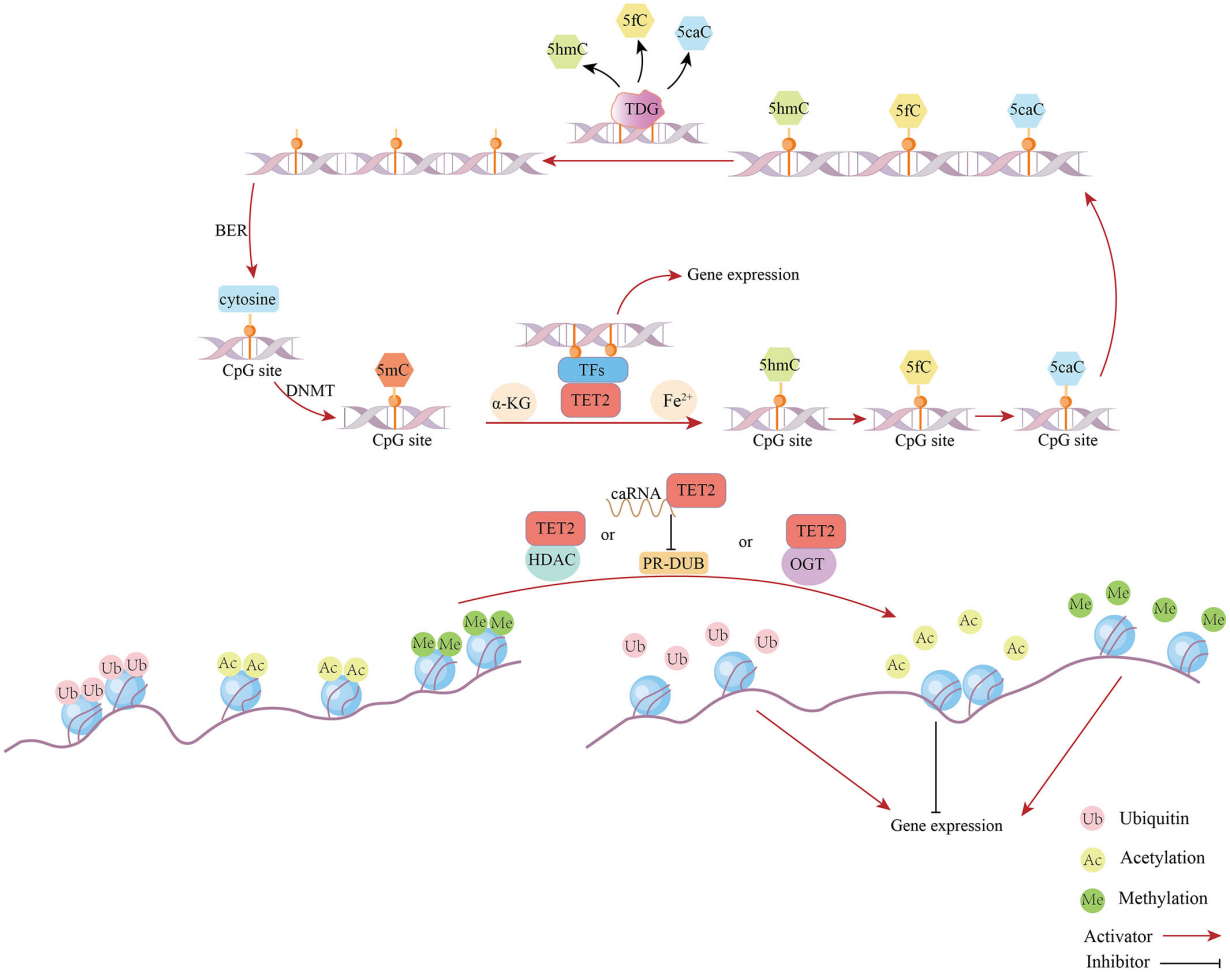
Pioneer transcription factors recruit TET2 to gene regulatory regions. With α-KG and ferrous iron serving as cofactors, TET2 initiates the active DNA demethylation cascade by catalyzing the stepwise oxidation of 5mC into 5hmC, 5fC and 5caC. These oxidized cytosine derivatives are recognized and cleaved by TDG, which generates abasic sites. Such lesions are subsequently repaired via the BER pathway to regenerate unmethylated cytosine, thereby accomplishing complete active DNA demethylation. By contrast, DNA methyltransferases (DNMTs) catalyze DNA methylation and reverse this epigenetic process. In addition, TET2 interacts with and recruits HDACs, PR-DUB complexes and OGT to jointly regulate histone modifications. Specifically, HDACs recruited by TET2 mediate histone deacetylation, which generally represses gene transcription. Conversely, TET2 forms a stable complex with OGT to drive histone demethylation at chromatin. In parallel, TET2 modulates the oxidation of m^5^C residues on caRNAs, which impedes PR-DUB complex recruitment. Collectively, these events remodel chromatin into an open conformation and facilitate transcriptional activation of downstream target genes.

TET2 exerts pleiotropic effects critical for hematopoietic stem cell (HSC) self-renewal, differentiation, genomic stability, and inflammatory resolution (10–13). Single-cell genomic analyses reveal TET2 targeting of gene promoters and transcription factor binding sites, where dysregulated methylation profoundly alters cell fate and contributes to disease pathogenesis (10, 14, 15). Somatic mutations within the *TET2* gene are prevalent in hematopoietic cancers, including myeloid and lymphoid malignancies, and are also identified in certain solid tumors (16). TET2-mediated epigenetic modifications, particularly those involving 5hmC, are essential for gene regulation in both cancer and immune cells. In oncogenesis, perturbed TET2 function frequently correlates with a distinct DNA methylation pattern characterized by global hypomethylation punctuated by localized promoter hypermethylation. Beyond its canonical DNA demethylation function, TET2 exhibits broad epigenetic regulatory functions through associations with various epigenetic modifiers, including histone deacetylase (HDAC), O-linked N-acetylglucosamine transferase (OGT) complexes formation and chromatin-associated RNAs (caRNAs) epitranscriptomic modifications. Mechanistically, TET2 recruits HDAC2 to drive histone deacetylation, thereby repressing the transcription of pro-inflammatory interleukin (IL)-6 during inflammation resolution (13). Meanwhile, TET2 forms a stable complex with OGT to synergistically modulate histone demethylation (17). In addition, TET2-mediated oxidation of m^5^C on caRNAs hinders methyl-CpG-binding domain protein 6 (MBD6)-dependent recruitment of the PR-DUB complex, inhibits monoubiquitinated Lys119 of histone H2A (H2AK119ub) deubiquitination, and further regulates chromatin accessibility and global gene transcription levels (**Figure 1**) (18). The intricate link between epigenetic dysregulation and CVD is an expanding field, with TET2 dysfunction specifically implicated in atherosclerosis. Moreover, TET2’s involvement in neurological and metabolic disorders is garnering increasing attention.

**Figure 2:**
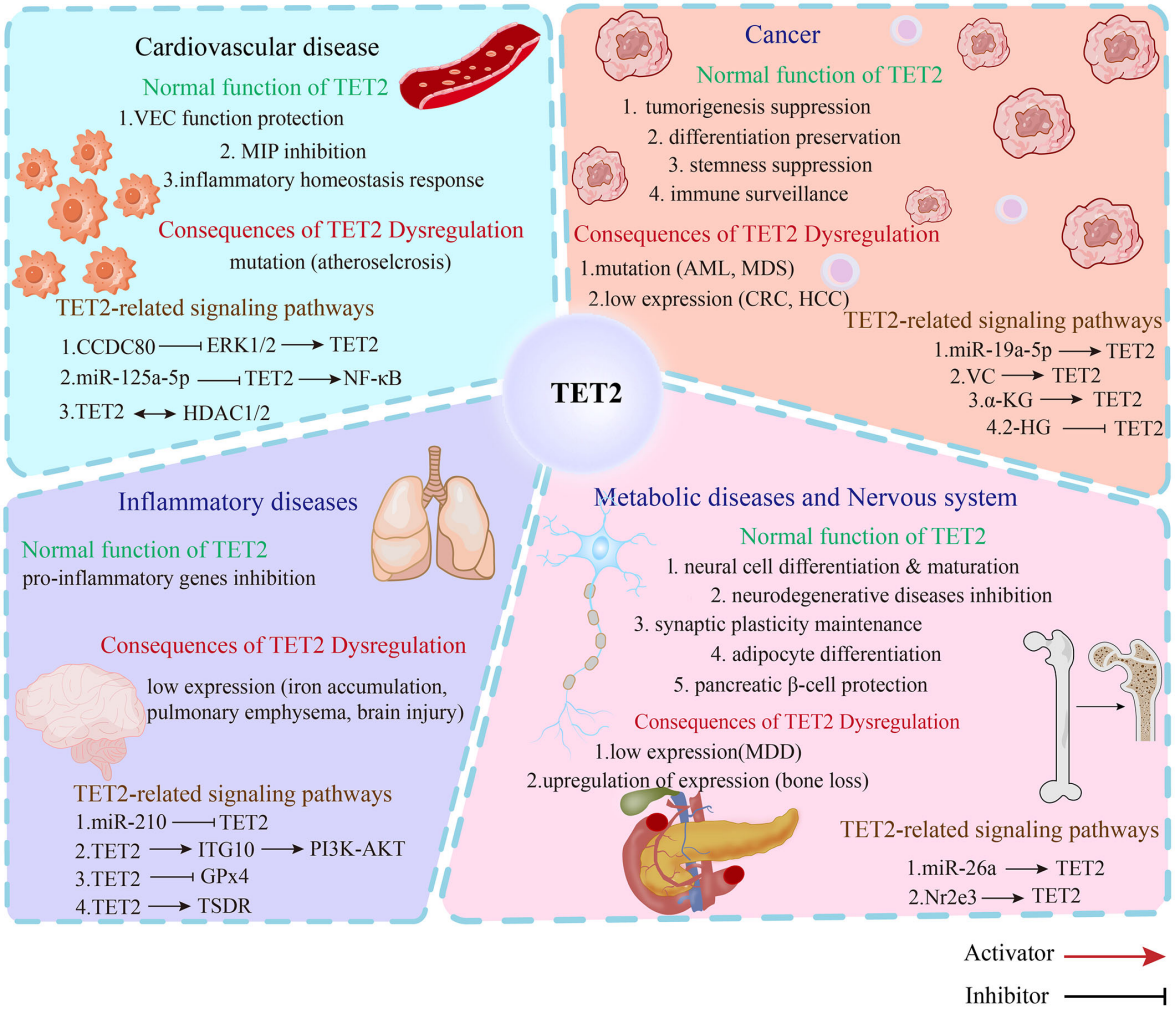
The regulatory roles of TET2 in various diseases. This diagram delineates the normal functions of TET2 and the consequences of its dysregulation across CVD, inflammatory diseases, cancer, metabolic diseases, and the nervous system. This figure highlights TET2’s critical roles and intricate molecular pathways, demonstrating its significant impact on health and disease. In the figure, arrows in red denote promotion, and T-shaped symbols in black denote inhibition.

Departing from the traditional focus on certain disease phenotypes, this review provides a systemic integration of TET2’s multi-dimensional network as a central epigenetic hub across oncological, inflammatory, and metabolic systems. By elucidating both its catalytic and non-catalytic roles, we highlight the critical context-dependency that dictates its divergent functions within different disease environments. Given the rapid clinical advancement of isocitrate dehydrogenase (IDH) inhibitors and metabolic interventions, we establish a unified framework to bridge the current gap between molecular mechanisms and therapeutic strategies. Ultimately, this synthesis provides a theoretical roadmap for precision targeting, underscoring TET2’s potential to resolve the shared pathophysiology of complex human diseases, as shown in **Figure 2**.

## 2 Structure of TET2

**Figure 3:**
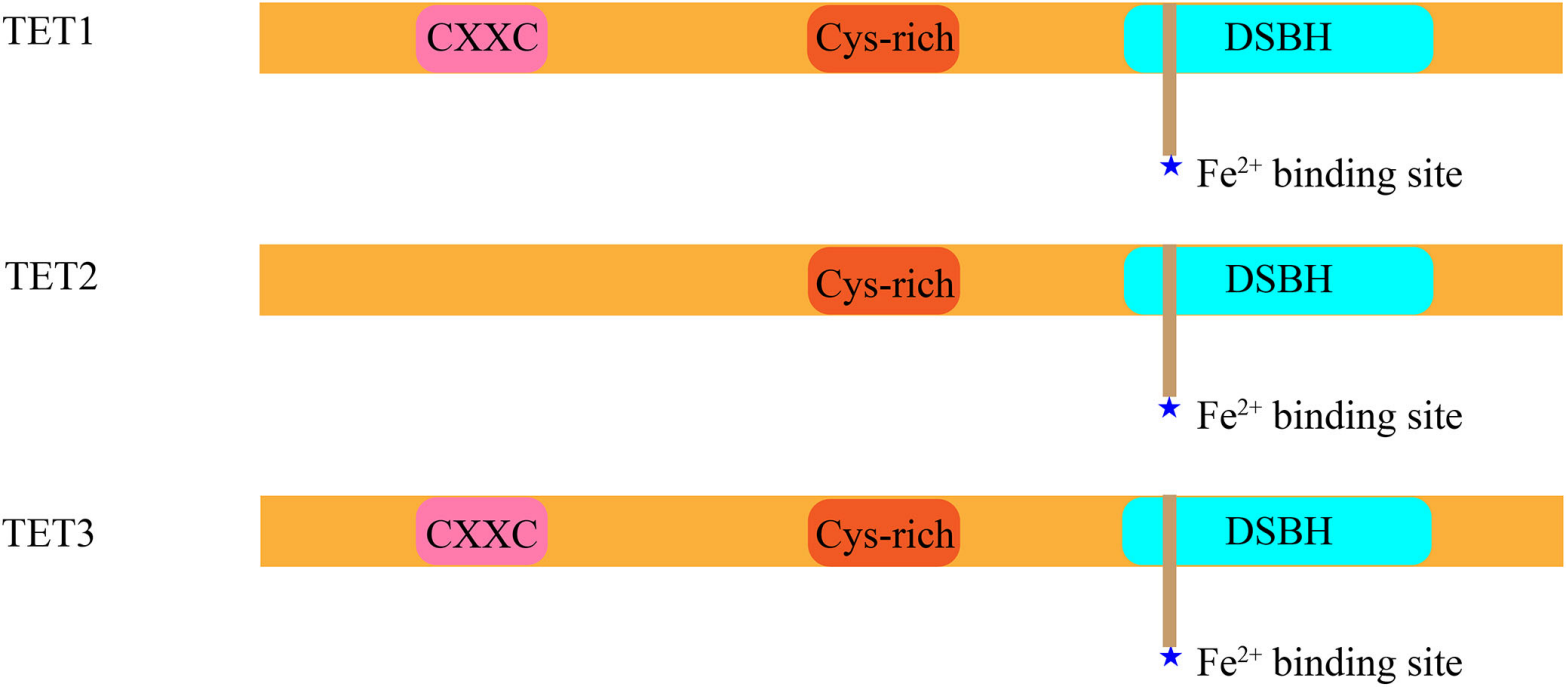
The schematic illustration of structural domain organization of the TET protein family. This diagram illustrates the conserved and divergent structural features of the TET protein family members. Each TET enzyme (TET1, TET2, and TET3) comprises a catalytic core, which includes a Cys-rich domain and a DSβH domain. The DSβH domain, containing binding sites for Fe^2+^ and α-KG (indicated by asterisks), mediates the dioxygenase activity. Notably, full-length TET1 and TET3 proteins possess an N-terminal CXXC zinc finger domain, which mediates DNA binding. In stark contrast, TET2 is distinguished by the absence of this CXXC DNA-recognition domain, a key structural difference that influences its genomic targeting and regulatory functions.

The TET protein family comprises three members: TET1, TET2, and TET3 (19). These enzymes are central to maintaining the fidelity of DNA methylation patterns through their catalytic involvement in demethylation (19). The dioxygenase domains of TET1, TET2, and TET3 consist of a cysteine-rich (Cys-rich) domain and a tightly packed double-stranded β-helix (DSβH) fold domain (20). Within the DSβH domain, which mediates the dioxygenase activity, there are three Fe^2+^ binding sites and one α-KG binding site essential for catalysis (20). Unique to TET1 and TET3, a CXXC-type zinc finger domain is present at the N-terminus. This CXXC domain, characterized by its Zn^2+^ coordination, forms a structural motif capable of binding to specific DNA/RNA sequences, thereby dictating the genomic targeting of TET1 and TET3. In stark contrast, TET2 lacks this CXXC DNA recognition domain; therefore, it must collaborate with DNA-binding proteins such as transcription factors to regulate DNA methylation. Dominant epigenetic pioneer transcription factors (TFs) recruit TET2 to regulatory regions, and by reshaping the distribution patterns of 5mC and 5hmC, they modulate the accessibility of key transcription factors to their genomic binding motifs (20, 21). The human *TET2* gene is located on chromosome 4q24, spans approximately 150 kb, and consists of 11 exons (2, 22). Its structural divergence, specifically the absence of the cytosine-phosphate-guanine (CpG) DNA-binding CXXC domain, is attributed to a gene splitting event that occurred during vertebrate evolution (2). Here, we use the schematic illustration to summarize the basic structure of TET family members in **Figure 3**.

## 3 Regulation of TET2 in cancer

TET2 dysfunction in cancer, driven by *TET2* mutations and aberrant regulation of its expression (21), precipitates profound alterations in DNA methylation and gene expression—hallmarks of oncogenesis (23). A consistent feature in malignant transformation is a pervasive redistribution of DNA methylation, often characterized by global hypomethylation juxtaposed with focal hypermethylation at gene promoter regions (24). Analysis of the COSMIC database reveals specific *TET2* mutations (including only frameshift, nonsense, and missense mutations) are predominantly observed in hematologic malignancies, accounting for 71% (2,074/2,923) of all reported cases (25). Among these, 65% are associated with myeloid lineage diseases and 35% with lymphoid system disorders (25). Notably, within hematologic malignancies, mutations significantly cluster around the oxygenase domain of *TET2*, exhibiting an approximately equal distribution across the three mutation types (25). In contrast, only 29% (849/2,923) of these mutations are reported in solid tumors. Over 75% of *TET2* mutations in solid tumors are missense mutations, predominantly documented as single-case reports (25). It is therefore postulated that many of these solid tumor mutations may represent neutral, passenger, or low-frequency germline variants, with their specific oncogenic functions remaining largely uncharacterized.

### 3.1 Hematological tumors

#### 3.1.1 Regulatory role of TET2 in myeloid malignancies

Myeloid malignancies, encompassing acute myeloid leukemia (AML), myelodysplastic syndromes (MDS), and myeloproliferative meoplasms (MPN), originate from clonal aberrations within the hematopoietic myeloid lineage (26–28). TET2 functions as a critical regulator of physiological hematopoiesis, particularly myelopoiesis. Functional loss of TET2 precipitates progressive hematopoietic defects, marked by enhanced self-renewal of HSCs and myeloid expansion (29). These observed phenotypes correlate with diminished 5hmC levels, widespread DNA hypermethylation in hematopoietic stem and progenitor cells (HSPCs), and altered gene expression profiles consistent with an inherent myeloid lineage bias in self-renewing progenitors (30).

**Figure 4:**
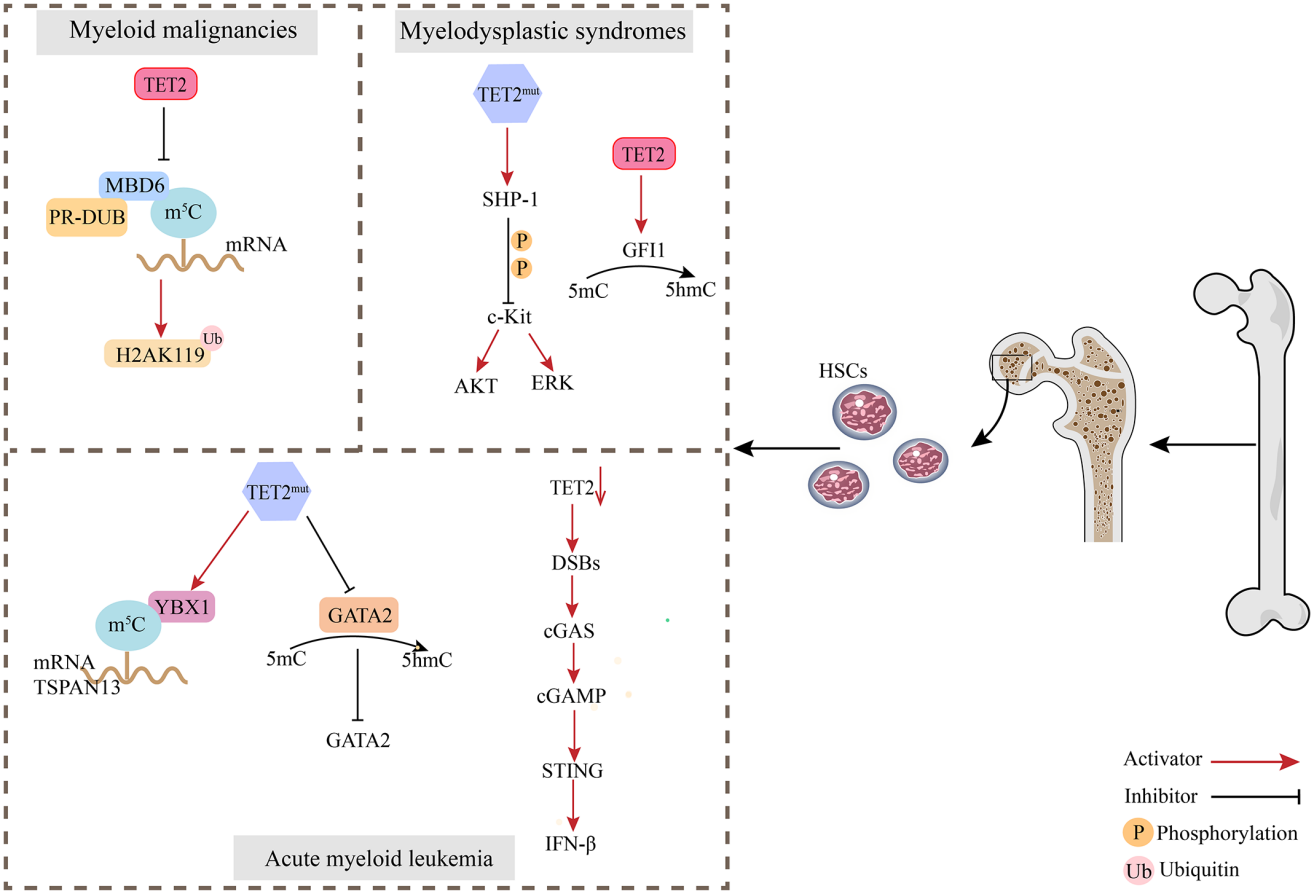
In myeloid malignancies, wild-type TET2 oxidizes m^5^C modifications on caRNAs, blocks the binding of MBD6 to m^5^C and hinders the recruitment of PR-DUB complexes. This maintains the stable abundance of H2AK119ub and retains chromatin in a compact, condensed conformation. In MDS, functional TET2 mediates the demethylation of *GFI1* to sustain normal hematopoietic homeostasis. In contrast, mutant *TET2* suppresses the activity of SHP-1, abolishes its inhibitory effect on phosphorylated c-Kit, and thereby persistently activates the downstream oncogenic AKT and ERK signaling cascades. In AML, loss of TET2 leads to massive accumulation of m^5^C modifications on *TSPAN13* mRNA. These methylated sites are specifically bound and recognized by YBX1 protein, which drastically stabilizes TSPAN13 transcripts and upregulates its expression. In addition, loss-of-function mutations in TET2 induce abundant deposition of methyl modifications at the promoter region of the *GATA2* gene, resulting in promoter hypermethylation and downregulated GATA2 expression, thereby relieving the growth-inhibitory effect of GATA2 protein on leukemic cells. Loss-of-function TET2 also leads to widespread accumulation of cellular DSBs, activating the cGAS-cGAMP-STING innate immune pathway and triggering the production of IFN-β. Meanwhile, oncogenic CEBPA-p30 protein binds the distal enhancer of *GATA2* gene and recruits TET2 to drive demethylation at the *GATA2* promoter, consequently upregulating GATA2 expression. In the figure, arrows denote promotion, and T-shaped symbols denote inhibition.

*TET2* mutations is one of the leading factors triggering the initiation and progression of myeloid malignancies. Chromatin-associated retrotransposon RNA containing m^5^C can be recognized by the MBD6 (18). MBD6, in turn, recruits PR-DUB complexes to catalyze the deubiquitination of H2AK119ub, thereby facilitating chromatin decompaction (31). Under normal conditions in adult cells, *TET2* maintains chromatin compaction by oxidizing m^5^C in caRNAs. However, TET2 loss in hematopoietic stem and progenitor cells lead to a global decrease in H2AK119ub, resulting in widespread chromatin opening, elevated transcription, and the aberrant reactivation of proliferation pathways (18). Critically, TET2 counteracts MBD6-dependent H2AK119ub deubiquitination by oxidizing m^5^C (18). This chromatin regulatory axis clarifies the dependency of *TET2*-mutant human leukemia on this specific gene activation pathway, as evidenced by MBD6 depletion in mouse models selectively impeding the proliferation of *TET2*-mutant leukemia cells and largely rescuing TET2 loss-induced hematopoietic defects (18). Consequently, this TET2-mediated oxidation of retrotransposon RNA m^5^C and its downstream effector MBD6 represent a promising therapeutic vulnerability in *TET2*-mutant malignancies (**Figure 4**).

#### 3.1.2 Regulatory role of TET2 in MDS

MDS represent a heterogeneous group of clonal myeloid disorders originating from hematopoietic stem cells, characterized by dysplastic myeloid differentiation, ineffective hematopoiesis, refractory cytopenias, and an elevated risk of progression to AML (32, 33). Anemia is a consistent manifestation of cytopenia in MDS patients (34). Heterozygous loss-of-function mutations in *TET2* are highly prevalent in MDS, and TET2 insufficiency profoundly impairs erythroid differentiation. This deficiency renders erythroid progenitors (CFU-E) abnormally dependent on erythropoietin (EPO) and stem cell factor (SCF), giving rise to a distinct “marked CFU-E” population (35). These aberrant cells display heightened c-Kit phosphorylation, reduced expression of the negative c-Kit regulator SHP-1 phosphatase, and elevated AXL tyrosine kinase expression, collectively driving sustained AKT and ERK pathway activation (35). This signaling imbalance promotes unchecked proliferation but concurrently blocks terminal erythroid differentiation, culminating in ineffective hematopoiesis and severe anemia.

In addition, studies have demonstrated that the zinc-finger repressor GFI1 restricts the proliferation of hematopoietic stem cells (HSCs) and preserves their functional integrity (36, 37). Loss of *GFI1* results in excessive proliferation and impaired repopulation capacity of HSCs (36, 37). TET2 sustains GFI1 expression by mediating DNA demethylation in the *GFI*1 promoter region. Impaired TET2 activity increases methylation levels of the *GFI1* promoter to downregulate GFI1, ultimately driving excessive expansion of hematopoietic stem cells (38).

#### 3.1.3 Regulatory role of TET2 in AML

With the widespread application of high-throughput sequencing technologies, the molecular classification of AML has been progressively refined, and mutations in epigenetic regulatory genes have been recognized as critical determinants shaping the AML genomic landscape (39–41). As a key epigenetic regulator, *TET2* encodes a DNA demethylase that catalyzes the oxidation of 5mC to 5hmC to initiate active DNA demethylation, which is essential for maintaining hematopoietic epigenetic homeostasis (26). Loss-of-function mutations in TET2 are among the most prevalent epigenetic driver alterations in adult AML (**Figure 4**) (42).

Traditional views hold that TET2 primarily regulates gene transcription through DNA demethylation. However, emerging studies have expanded its biological functions to multiple non-canonical dimensions, mainly including DNA damage immune response, post-transcriptional RNA modification, and inflammatory hematopoietic regulation. Notably, the discovery of these multi-dimensional functions has not only deepened the understanding of AML pathogenesis but also provided novel targets for precise therapeutic strategies based on *TET2* mutation status.

Specifically, *TET2* deficiency impairs the DNA damage repair capacity of HSPCs and increases genomic instability. Accumulated DNA double-strand breaks (DSBs) trigger the activation of cyclic GMP-AMP synthase (cGAS), a cytoplasmic DNA sensor (43). cGAS catalyzes the synthesis of the second messenger 2’,3’-cyclic-GMP-AMP (cGAMP), which further activates stimulator of interferon genes (STING) and initiates the transcription of type I interferon (IFN-β) (44). Activation of the cGAS-STING pathway endows TET2-deficient HSPCs with enhanced self-renewal capacity and facilitates the progression of clonal hematopoiesis (CH).

At the RNA level, in addition to its canonical function in regulating DNA methylation, TET2 serves as an RNA m^5^C demethylase that participates in post-transcriptional regulation. *TET2* deficiency induces massive accumulation of m^5^C modifications on tetraspanin 13 *(TSPAN13)* mRNA, which are specifically recognized by YBX1 and thereby markedly enhance the stability and expression abundance of *TSPAN13* transcripts (45). TET2-mediated mRNA m^5^C demethylation exerts crucial regulatory effects on hematopoiesis, leukemic stem cell (LSC) migration and homing, as well as LSC self-renewal.

*TET2* and *GATA2* frequently present co-mutations in CEBPA double-mutant AML (46). Elevated GATA2 inhibits AML cell survival, whereas loss-of-function *TET2* mutations counteract this aberrant *GATA2* upregulation to abolish its tumor-suppressive functions and maintain the malignant survival of leukemia cells (46).

### 3.2 Solid tumors

**Figure 5:**
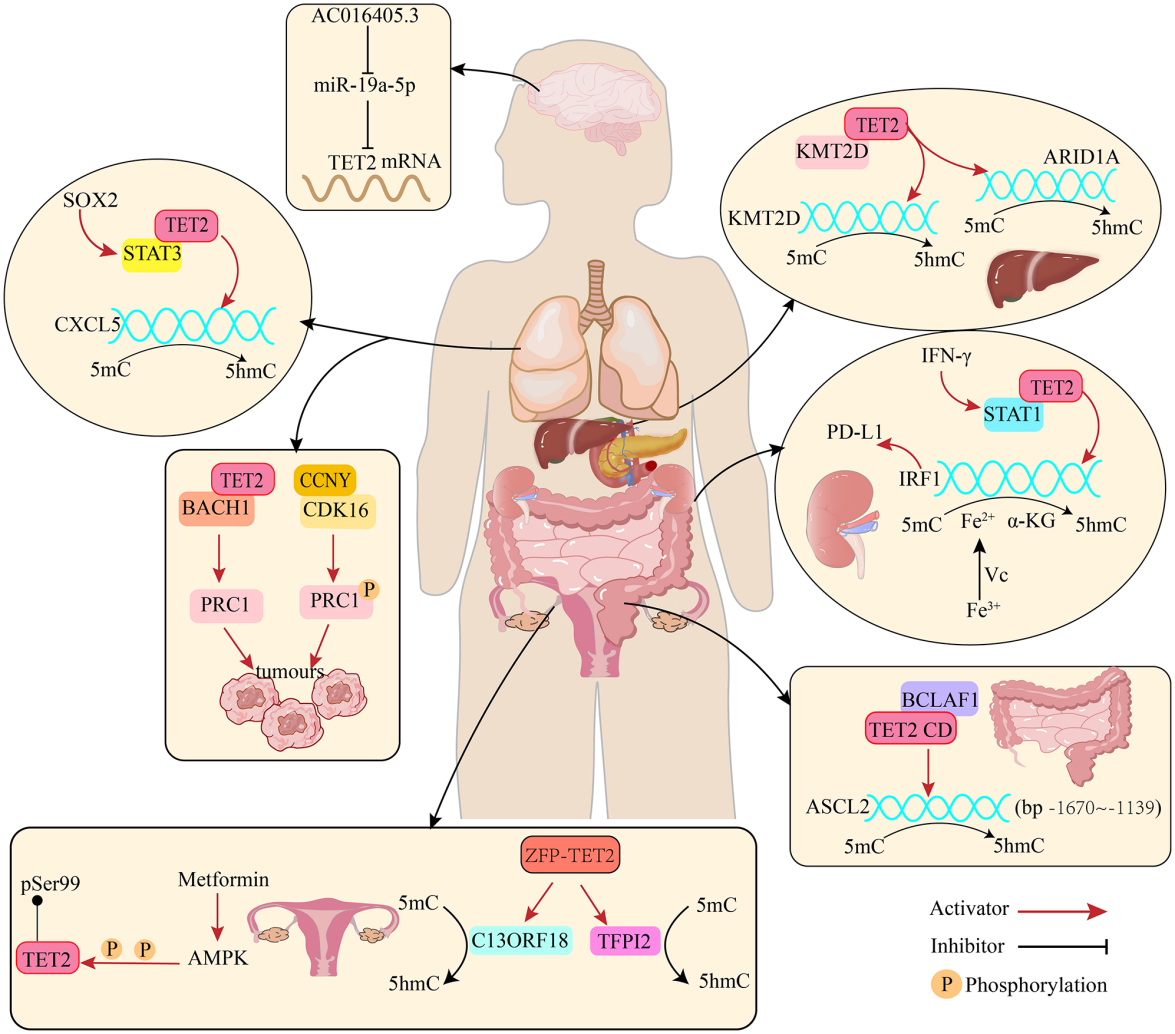
In GBM, the long non-coding RNA AC016405.3 acts as a competing endogenous RNA to sponge miR-19a-5p. It relieves the inhibitory effect of this microRNA on target genes, upregulates TET2 expression, and ultimately suppresses the proliferation, invasion and metastasis of GBM cells. In lung cancer, SOX2 activates STAT3, which subsequently recruits TET2 to the promoter of *CXCL5* and initiates the transcription of CXCL5. Another oncogenic signaling axis exists: TET2 separately binds BACH1 and the CCNY/CDK16 complex to form two distinct protein assemblies. These two complexes activate PRC1 and phosphorylated PRC1 respectively, synergistically facilitating tumorigenesis. In RCC, Vitamin C reduces ferric iron (Fe^3+^) to ferrous iron (Fe^2+^). Fe^2+^ together with α-ketoglutarate are essential cofactors for sustaining the catalytic activity of TET2. Upon IFN-γ stimulation, TET2 interacts with STAT1 to mediate demethylation of the *IRF1* promoter and upregulate PD-L1 expression. In HCC, KMT2D recruits TET2 to positively drive its own transcription. Meanwhile, TET2 recruited by KMT2D also triggers transcriptional activation of another target gene, *ARID1A*. In CRC, the BCLAF1-TET2 complex modulates the methylation and hydroxymethylation levels of the *ASCL2/TSS1500* promoter upstream of the transcription start site of *ASCL2*, which mediates aberrant overexpression of ASCL2. In CC, the complex formed by zinc finger protein ZFP and TET2 efficiently reverses promoter hypermethylation of tumor suppressor genes *TFPI2* and *C13ORF18*, and steadily restores their transcriptional expression. In the figure, arrows denote promotion, and T-shaped symbols denote inhibition.

In addition to hematological malignancies, TET2 has also been demonstrated to be abnormally expressed in various solid tumors and is involved in regulating cancer initiation and progression. We summarize the regulatory mechanisms of TET2 in multiple cancers, as illustrated in **Figure 5**.

#### 3.2.1 Regulatory role of TET2 in endometrial cancer

EC is a major gynecological malignancy, accounting for 20-30% of female reproductive tract malignancies and 7% of all female systemic cancers (47). While clinical risk factors of EC like obesity, type 2 diabetes, and PCOS are well-documented (48, 49), the molecular link lies in the regulatory dysfunction of the tumor suppressor TET2. TET2 serves as a pivotal metabolic-epigenetic sensor; its stability is regulated by the energy-sensing kinase AMPK, which phosphorylates TET2 at Serine 99 (Ser99) (50). This phosphorylation is a critical regulatory switch: it prevents TET2 degradation, allowing it to maintain the epigenetic landscape by catalyzing the conversion of 5mC to 5hmC. In EC cells, the activation of the AMPK-TET2 signaling axis—often via metformin—restores 5hmC levels, thereby re-establishing the transcriptional programs that suppress tumor proliferation (51). This restoration ultimately suppresses endometrial cancer cell proliferation, a mechanism demonstrably effective in TET2-dependent tumor models.

#### 3.2.2 Regulatory role of TET2 in lung cancer

Lung cancer, originating from the bronchial mucosa or glands of the lungs, remains a leading cause of global cancer incidence and mortality (52). Adeno-to-squamous transition (AST) in lung cancer represents a classic histological transformation associated with robust cancer plasticity – the ability to switch between different lineages (53). TET2-mediated epigenetic mechanisms govern this phenotypic shift, notably by promoting neutrophil infiltration and the transfer of lipids from neutrophils to cancer cells. Enhanced phenotypic plasticity is a growing hallmark of cancer, and increased neutrophil infiltration is frequently observed during AST, correlating with squamous lesions (54). Moreover, transient neutrophil depletion has been shown to impede squamous transformation, highlighting their role in this process (55).

CXCL5 has been identified as a crucial chemokine in neutrophil recruitment during AST. TET2 is recruited by signal transducer and activator of transcription (STAT) 3 to the *CXCL5* promoter region, thereby activating its transcription (53). The transcription factor sex-determining region Y-box 2 (SOX2) further amplifies this cascade by promoting STAT3 activation, which in turn upregulates *CXCL5* gene expression (56, 57). Additionally, STAT3 directly interacts with TET2 to modulate downstream inflammatory signaling. Thus, TET2 actively promotes neutrophil infiltration via STAT3-mediated CXCL5 axis. Targeting the STAT3-CXCL5 pathway has demonstrated efficacy in suppressing squamous transformation by attenuating neutrophil infiltration.

Beyond its regulatory role in atherosclerosis (AST), TET2 cooperates with the CNC-bZIP transcription factor family member BACH1 in non-small cell lung cancer (NSCLC) (58). It recruits BACH1 to the *PRC1* promoter, facilitates DNA demethylation, relieves chromatin repression, and thereby upregulates the transcription and expression of *PRC1*, while cyclin Y (CCNY), a key regulator of excessive PRC1 activation, forms an active complex with CDK16 to enhance PRC1 phosphorylation (58). This dual activation—transcriptional upregulation by TET2 and post-translational activation synergistically enhances spindle formation and cell cycle progression, thereby accelerating NSCLC cell proliferation, colony formation, and tumorigenic capacity both *in vitro* and *in vivo*.

In summary, TET2 participates in lung cancer progression through two independent signaling axes: the STAT3-CXCL5-neutrophil axis and the BACH1-PRC1-CCNY/CDK16 axis, which modulate histological plasticity and cell proliferation respectively (53, 58). These findings broaden the functional spectrum of TET2 in solid tumors and identify novel candidate targets for epigenetic targeted therapy against lung cancer.

#### 3.2.3 Regulatory role of TET2 in glioblastoma

In central nervous system, GBM is the most common and aggressive primary malignant tumor (59). Long non-coding RNAs (lncRNAs) are increasingly recognized as critical regulators in various malignancies, including GBM (60, 61). Research found a mutual inhibitory relationship between miR-19a-5p and the lncRNA AC016405.3, with miR-19a-5p functioning as a key downstream effector in AC016405.3-mediated proliferation and metastasis (62). Specifically, upregulation of miR-19a-5p counteracted the suppressive effects of AC016405.3 overexpression on GBM cell proliferation and metastasis (62). Notably, TET2 has been identified as a downstream target of miR-19a-5p, exhibiting a significant positive correlation with AC016405.3 expression (62). Therefore, AC016405.3 exerts its inhibitory effects on GBM cell proliferation and metastasis by sponging miR-19a-5p, which consequently leads to the upregulation of TET2.

#### 3.2.4 Regulatory role of TET2 in renal cell carcinoma

RCC is a prevalent malignancy of the genitourinary system, where the programmed death 1 (PD-1)/programmed death-ligand 1 (PD-L1) pathway has emerged as a cornerstone of its immunotherapy. However, the efficacy of these therapies is often hampered by significant individual variability in PD-L1 expression, driven largely by underlying epigenetic dysregulation (63). A central player in this regulatory landscape is the DNA hydroxylase TET2, which is frequently functionally impaired in RCC, leading to a profound reduction in 5hmC levels (64). TET2 acts as a critical mediator of the immune microenvironment by bridging metabolic status and gene expression. As an essential cofactor for Fe^2+^ and α-KG-dependent dioxygenases, Vitamin C specifically stimulates TET2 activity, thereby restoring the 5hmC profile and remodeling the transcriptome of RCC cells (64, 65).

Mechanistically, this regulatory role is essential for immune recognition. Murine models further demonstrate that TET2 deficiency impairs interferon (IFN)-γ-induced chemokine and PD-L1 expression, consequently reducing lymphocyte infiltration within the RCC microenvironment (66, 67). Beyond its general activating effect on TET2 in normal contexts, Vitamin C specifically enhances TET2 enzymatic activity in RCC. TET2 is recruited to interferon regulatory factor 1 (IRF1) via IFN-γ-STAT1 signaling, maintaining IRF1 in a demethylated state, ultimately inducing PD-L1 expression (67). Comparative studies reveal that Vitamin C treatment significantly augments intratumoral T cell infiltration relative to vehicle or anti-PD-L1 monotherapy, whereas TET2 ablation profoundly compromises T cell infiltration (67).

#### 3.2.5 Regulatory role of TET2 in hepatocellular carcinoma

HCC is the predominant subtype of primary liver malignancy and a major contributor to cancer-related mortality worldwide (68). Research shows that DNA dioxygenase TET2 collaborates with histone-lysine N-methyltransferase 2D (KMT2D) to promote the transcription of KMT2D and AT-rich interaction domain 1A (ARID1A) in HCC (69). The catalytic domain (CD) of TET2 directly interacts with the PHD4–6 domains of KMT2D (69). This interaction enables KMT2D to recruit TET2, thereby promoting its own transcriptional activation. Similarly, ARID1A, another potential target, is also transcriptionally activated when KMT2D recruits TET2 (69, 70). KMT2D plays a pivotal role in mediating the anti-tumor effects of Vitamin C, a known TET2 agonist, in HCC. Notably, in terms of chemotherapy response, KMT2D deficiency renders HCC tumors more susceptible to cisplatin chemotherapy (69).

#### 3.2.6 Regulatory role of TET2 in colorectal cancer

CRC have identified specific genes and proteins that sustain stemness within CRC cell populations, including CRC progenitor cells (71). Achaete-scute homolog 2 *(ASCL2)*, a basic helix-loop-helix transcription factor, is implicated in CRC tumorigenesis (72, 73). Aberrant *ASCL2* upregulation is linked to hypermethylation within a specific region of its promoter (bp -1670~-1139) (74). Studies found that TET2-mediated *ASCL2* expression depends on the TET2 CD (74). The TET2 CD can bind endogenous B-cell lymphoma-2-associated transcription factor 1 (BCLAF1). TET2 directly binds to the N-terminal zinc finger domain of BCLAF1 via its C-terminal catalytic domain. The BCLAF1-TET2 complex regulates *ASCL2* overexpression in CRC cells by mediating the hydroxymethylation and methylation levels at the *ASCL2/TSS1500* promoter (74).

#### 3.2.7 Regulatory role of TET2 in cervical cancer

CpG hypermethylation-mediated epigenetic silencing of multiple tumor suppressor genes critically drives CC tumorigenesis (75, 76). Nevertheless, current epigenetic therapeutic strategies have notable limitations. The broad-spectrum demethylating agent 5-Aza-2’-deoxycytidine (5-aza-dC) lacks genomic targeting specificity and causes severe systemic toxicity, restricting its long-term use in solid tumor treatment (76, 77). Moreover, artificial transcriptional activators including ZFP-VP64 only transiently upregulate target genes without erasing aberrant epigenetic methylation marks, yielding short-term regulatory effects (78).

TET2 is a core enzyme of active DNA demethylation, which initiates epigenetic restoration by oxidizing 5mC and triggering downstream demethylation repair cascades. Cutting-edge epigenetic editing systems such as ZFPs, TALEN and CRISPR-Cas9 system enable precise recruitment of effector proteins to specific gene promoters for locus-specific epigenetic modulation. Based on this principle, many fusion epigenetic editors have been constructed for targeted epigenetic remodeling (79–83). Notably, in CC, ZFP-TET2 efficiently reverses promoter hypermethylation of the tumor suppressor genes *TFPI2* and *C13ORF18* to restore their transcriptional expression, thereby inhibiting malignant proliferation and reducing apoptosis resistance of CC cells (79). Its dual strengths of targeting accuracy and long-term remodeling capacity render ZFP-TET2 a promising tool for precision epigenetic gene therapy against CC.

## 4 The role of TET2 in inflammation

**Figure 6:**
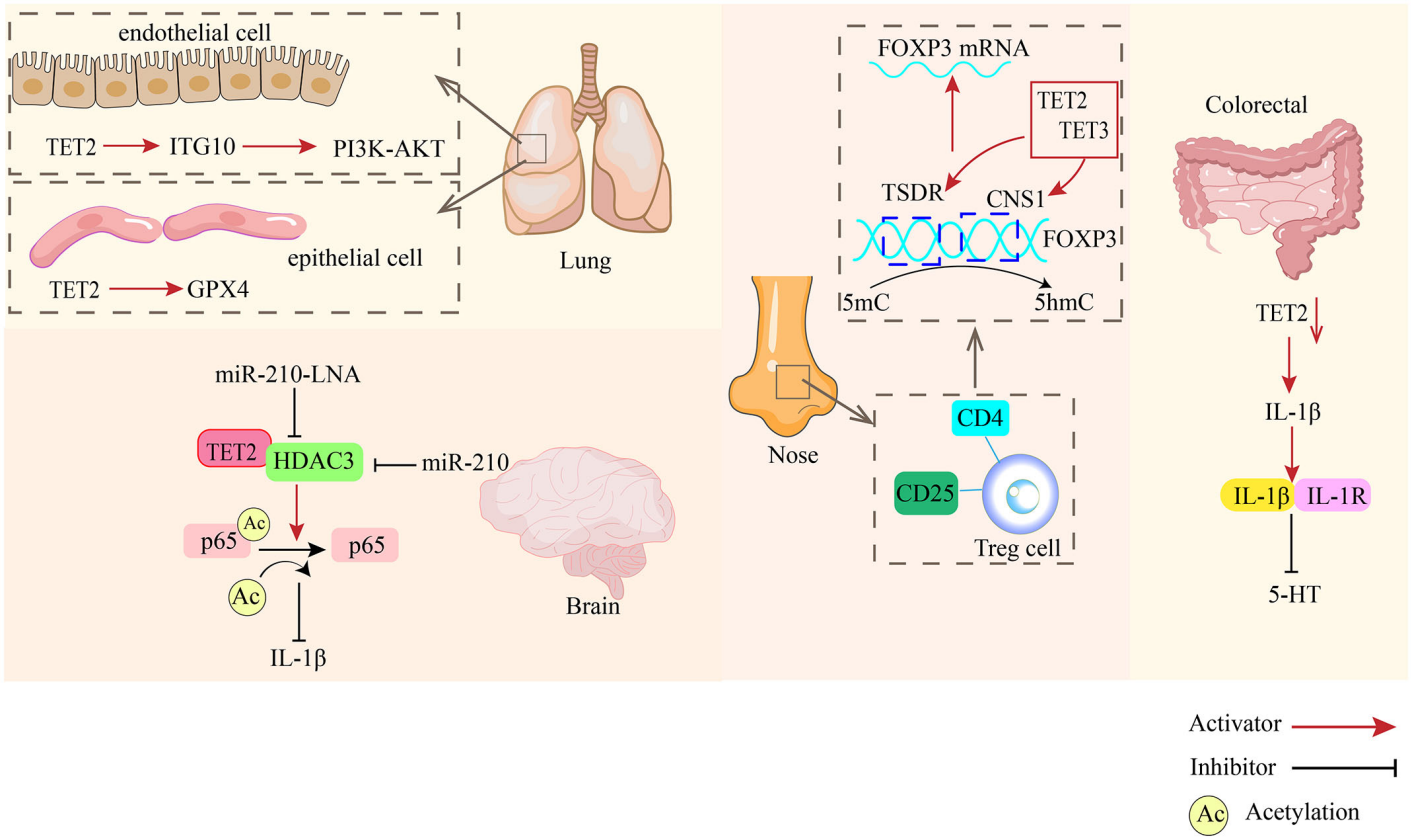
The dual regulatory roles of TET2 in inflammatory diseases. In ALI, TET2 upregulates ITGA10 to activate the PI3K-AKT pathway, protecting endothelial cells. ITA acts as an inhibitor of TET2. In COPD, TET2 alleviates cigarette smoke-induced ferroptosis in airway epithelial cells by demethylating the *GPX4* promoter. In AR, TET2 and TET3 jointly promote demethylation at the *TSDR* region to sustain FOXP3 expression and the function of Tregs. In brain injury, the miR-210/TET2/HDAC3 axis regulates NF-κB p65 acetylation and IL-1β expression, influencing neuroinflammation. In AKI, TET2 modulates metabolic and inflammatory responses via the PPAR pathway. In intestinal inflammation, myeloid-specific knockout of *TET2* leads to robust secretion of IL-1β. This cytokine binds to IL-1R on TH-positive catecholaminergic neurons, blocks signal communication between sympathetic neurons and intestinal epithelial cells, downregulates α1-adrenergic signaling, and reduces the levels of 5-HT. In the figure, arrows denote promotion, and T-shaped symbols denote inhibition.

TET2 is also a key epigenetic regulator in inflammatory responses. Its DNA demethylase activity enables the dynamic regulation of the expression of key genes involved in immune and inflammatory pathways. However, the role of TET2 is context-dependent: like a double-edged sword, it can either exert a protective effect or exacerbate pathological processes under different disease states. The relevant findings regarding acute lung injury, chronic obstructive pulmonary disease, acute kidney injury, allergic rhinitis, and brain injury are summarized as illustrated in **Figure 6**.

### 4.1 The role of TET2 in acute lung injury

ALI is a severe inflammatory lung disorder triggered by various factors such as infection, trauma, and sepsis. Its core pathological characteristics encompass an overwhelming inflammatory response, extensive damage to alveolar epithelial and vascular endothelial cells (VECs), neutrophil infiltration, and alveolar edema. In severe cases, it can progress to acute respiratory distress syndrome (ARDS) (84, 85).

Human pulmonary microvascular endothelial cells (HPMECs) are key effector cells in sepsis-induced ALI. Both *in vitro* and *in vivo* models of sepsis-induced ALI consistently demonstrate significant reductions in TET2 and 5hmC levels. Conversely, *TET2* knockdown exacerbates lipopolysaccharide (LPS)-induced HPMEC dysfunction and apoptosis, while TET2 overexpression significantly alleviates LPS-induced HPMEC dysfunction and reduces apoptosis (86). Mechanistically, TET2 overexpression reduces the methylation level of the integrin α10 *(ITGA10)* promoter, thereby increasing the expression of ITGA10 (86). The upregulated ITGA10 further activates the phosphatidylinositol 3-kinase (PI3K)-AKT signaling pathway, ultimately exerting a protective effect against ALI (86).

Another related study showed that itaconate (ITA) alleviates the excessive inflammatory response by inhibiting TET2, thereby reducing its demethylation-dependent activation of pro-inflammatory genes. *IRG1* encodes aconitate decarboxylase 1 (ACOD1), a mitochondrial metabolic enzyme that catalyzes the decarboxylation of cis-aconitate to produce the anti-inflammatory metabolite ITA (87, 88). Although both ITA and α-KG are dicarboxylic acids sharing 4- or 5-carboxylates, crucial for TET2 co-substrate activity, ITA structurally lacks the C-1 carboxylate and C-2 keto groups necessary for effective Fe^2+^ binding (89). This structural difference enables ITA to function as a competitive α-KG antagonist, thereby inhibiting TET2 and related dioxygenases. LPS, a classic inducer of sepsis, significantly induces *IRG1* expression, leading to massive accumulation of ITA and subsequent inhibition of TET2 activity in macrophages (89). Thus, TET2 is a direct molecular target through which ITA exerts its anti-inflammatory effects in suppressing LPS-induced inflammatory gene expression.

### 4.2 The role of TET2 in chronic obstructive pulmonary disease

COPD is a progressive disorder primarily triggered by cigarette smoke (CS), characterized by irreversible airflow limitation (90). Oxidative stress induced by CS is accompanied by chronic inflammation and epigenetic alterations, such as changes in DNA methylation (91). Furthermore, accumulating evidence has demonstrated that ferroptosis plays a critical role in the pathogenesis of COPD (92). Notably, the expression of TET2 protein is significantly downregulated in airway epithelial tissues from COPD patients, murine models of COPD, and bronchial epithelial cells treated with cigarette smoke extract (CSE) (91). Subsequent experiments using *TET2* knockout (KO) mice have further revealed that TET2 can alleviate CS-induced ferroptosis in airway epithelial cells of COPD patients by mediating the demethylation of the glutathione peroxidase 4 *(GPX4)* gene, thereby regulating CS-elicited lipid peroxidation. Loss of TET2 function can induce ferroptosis, which in turn exacerbates CS-induced airway remodeling, inflammatory responses and emphysematous lesions (91). In CSE-treated airway epithelial cells, *TET2* silencing promotes ferroptosis, whereas its overexpression effectively inhibits this process (91). Collectively, these findings suggest that TET2 exerts a central role in regulating lipid peroxidation and ferroptosis in airway epithelial cells, highlighting its potential as a therapeutic target for CS-related COPD.

### 4.3 The role of TET2 in acute kidney injury

AKI constitutes a significant global health burden, affecting approximately 13.3 million patients annually (93). Both the initiation and maintenance phases of AKI are marked by robust inflammatory responses and leukocyte recruitment during endothelial and tubular cell injury (94). Cisplatin, a widely used chemotherapeutic agent, unfortunately exhibits nephrotoxicity, largely due to its preferential accumulation in proximal tubule segments. Mounting evidence now firmly establishes a critical role for inflammation in the pathogenesis of cisplatin-induced AKI (95–99).

In a cisplatin-induced mouse model, *TET2* KO was associated with alterations in metabolic pathways including retinol, arachidonic acid, linolenic acid metabolism, and the peroxisome proliferator-activated receptor (PPAR) signaling pathway (94). Consistent with this, *TET2* expression is also downregulated in other AKI mouse models and clinical samples (94). Collectively, these findings strongly suggest that TET2 confers renoprotective effects during AKI by intricately modulating both metabolic and inflammatory responses through the PPAR signaling pathway.

### 4.4 The role of TET2 in allergic rhinitis

Allergic diseases such as allergic rhinitis (AR) are generally characterized by the imbalance of T cell subsets and dysregulated expression of key transcription factors (100). Forkhead box P3 (FOXP3) acts as the master transcription factor of CD4^+^/CD25^+^ regulatory T cells (Tregs), governing the differentiation, phenotypic stability and immunosuppressive capacity of Tregs (101, 102). Three conserved non-coding sequence (CNS) regulatory elements, CNS1-3, regulate FOXP3 expression during Treg differentiation (103). Among these elements, intronic CNS2, also termed the Treg-specific demethylated region (TSDR), critically modulates the stability of FOXP3 expression via its DNA methylation status (104). Elevated methylation of CNS2 during cell proliferation is accompanied by FOXP3 downregulation, subsequently triggering phenotypic instability and impaired immunosuppressive function of Tregs (104). TET2 and TET3 work synergistically to mediate active DNA demethylation at the CNS1 and CNS2 loci of the *FOXP3* gene in natural Tregs (nTregs); simultaneous deletion of the two enzymes markedly impairs FOXP3 expression stability (105, 106). Treatment with Vitamin C, a TET activator, promotes the loss of 5mC and accumulation of 5hmC at CNS1/CNS2 sites, restores TSDR demethylation and sustains persistent FOXP3 expression (106, 107). In addition, IL-2, a critical cytokine for Treg homeostasis, maintains stable FOXP3 expression by upregulating TET2 (108). Loss of IL-2 suppresses TET2-dependent TSDR demethylation and leads to fluctuating FOXP3 levels (108). During nTreg development, IL-2 facilitates the upregulation of TET2, thereby sustaining steady FOXP3 expression (108).

### 4.5 The role of TET2 in brain injury

Neonatal hypoxic-ischemic (HI) brain injury is a leading cause of acute neonatal death and chronic disability. Under normal conditions, TET2 recruits HDAC3, which binds to the nuclear factor κB (NF-κB) subunit p65 and persistently suppresses p65 acetylation, reducing the expression of pro-inflammatory factors like IL-1β (13). Murine models of HI injury demonstrate a significant upregulation of miR-210, concomitant with a reduction in TET2 protein abundance. This leads to increased acetylation of NF-κB subunit p65 and enhanced DNA binding to the *IL-1β* promoter in the immature mouse brain (109–111). Furthermore, *TET2* knockdown exacerbated brain infarct volume and neurological impairments, simultaneously abrogating the neuroprotective effects of miR-210 inhibition. Conversely, inhibiting miR-210 rescued TET2 protein levels after HI injury (109–111). Suppressing cerebral miR-210 using a complementary locked nucleic acid oligonucleotide (miR-210-LNA) mitigated the ischemia stroke-induced inflammatory response in the adult mouse brain and provided neuroprotection for the immature rat brain after HI injury (109).

### 4.6 The role of TET2 in intestinal inflammation

The intestinal lamina propria is enriched with tissue-resident macrophages and other myeloid cells, which coordinate local inflammatory responses and serve as the central hub for immune interactions among neurons, epithelial cells, and stromal cells (112–114). Existing cellular experiments have demonstrated that loss of TET2 function reshapes the cytokine secretion profile of myeloid cells (e.g., increased IL-1β), suggesting that myeloid TET2 may participate in the dynamic regulation of intestinal inflammation by modulating immune-neuronal signaling crosstalk (115, 116). In a dextran sulfate sodium (DSS)-induced murine colitis model, myeloid-specific deletion of TET2 triggers massive IL-1β release; this cytokine binds to the IL-1 receptor on tyrosine hydroxylase (TH)-positive catecholaminergic neurons, suppressing sympathetic neuron-epithelial interactions, downregulating α1-adrenergic signaling, impeding enterochromaffin (EC) cell differentiation, and reducing 5-hydroxytryptamine (5-HT, also known as serotonin) levels, ultimately alleviating intestinal mucosal damage (116). These findings confirm that TET2 is a key epigenetic regulatory molecule integrating immune-neuronal signals to modulate intestinal inflammation and maintain the dynamic balance of neuro-epithelial signaling under physiological homeostasis.

## 5 The role of TET2 in CVD

CVD constitute the leading cause of global mortality, frequently driven by atherosclerosis of the coronary, cerebral, or peripheral arteries. Atherosclerosis is triggered by the vascular accumulation of cholesterol-rich lipoproteins, which undergo modification and subsequent uptake by macrophages, thereby triggering a chronic inflammatory response within the arterial wall (117).

**Figure 7:**
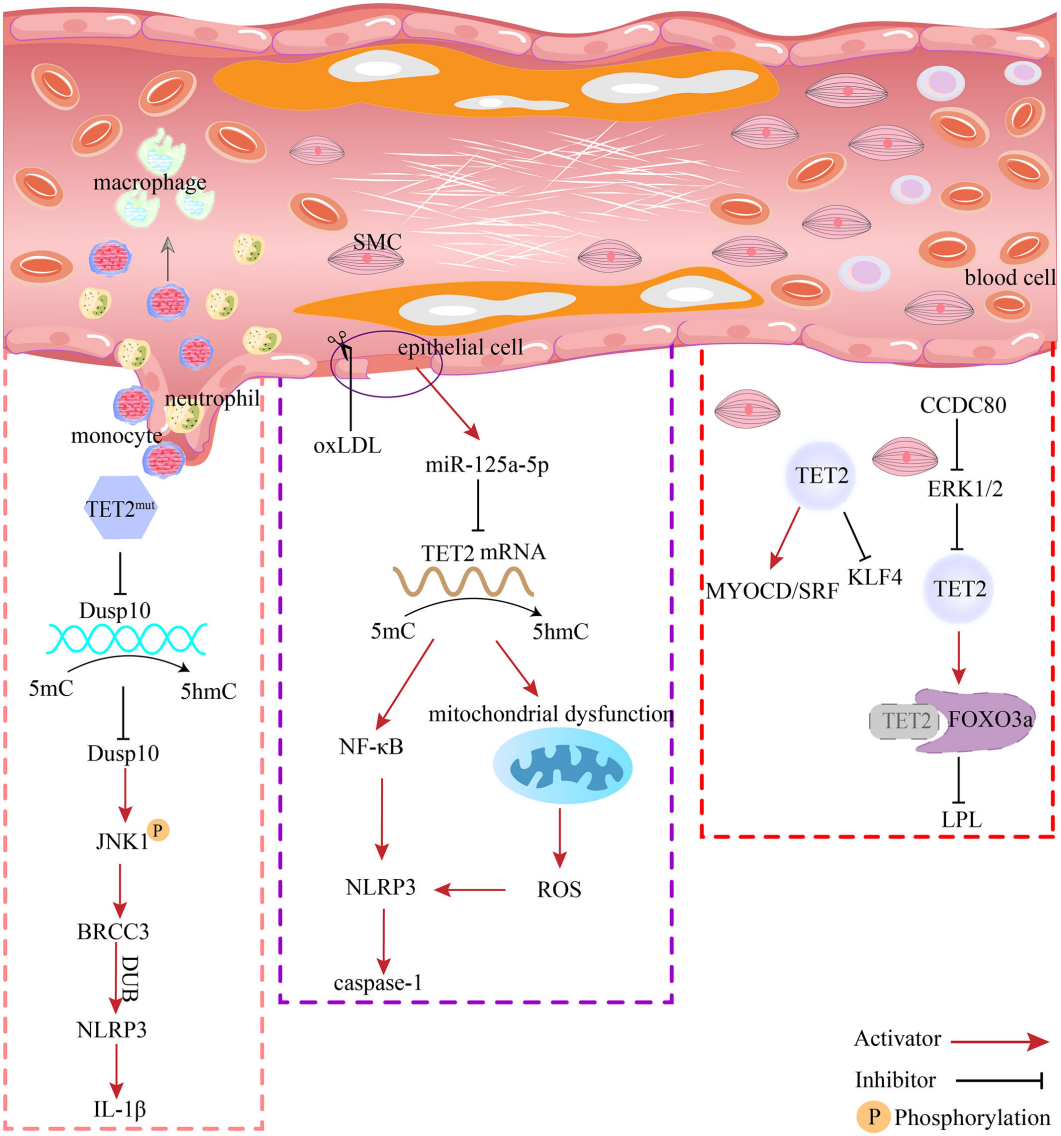
Mechanistic pathways of TET2 in atherosclerosis development and resolution. This schematic delineates the multifaceted roles of TET2 in regulating inflammation and SMC differentiation in atherosclerosis. *TET2* mutations enhance IL-1β expression through NLRP3 inflammasome activation. In hematopoietic cells, TET2 deficiency induces hypermethylation of the promoter of *DUSP10*, thereby driving JNK1 phosphorylation. This, in turn, activates the NLRP3 inflammasome through BRCC3-mediated deubiquitination. Regarding SMC plasticity, TET2 modulates MYOCD/SRF and KLF4 pathways, thereby fostering a contractile SMC phenotype and protecting against vascular injury. Reduced TET2 expression, influenced by CCDC80 and concomitant ERK1/2 inhibition, leads to increased DNA methylation and impaired FOXO3a interactions at the *LPL* promoter, thus exacerbating atherosclerosis. Moreover, through its targeting of TET2 mRNA, endogenous miR-125a-5p exacerbates mitochondrial dysfunction and thereby promotes the activation of NF-κB induced by oxLDL. This cascade leads to ROS accumulation and subsequent NLRP3 inflammasome activation, resulting in VEC pyroptosis and inflammation during atherogenesis. In the figure, arrows denote promotion, and T-shaped symbols denote inhibition.

TET2 directly binds to and recruits HDAC1/2 in an enzyme activity-independent manner. This interaction promotes histone deacetylation, thereby suppress the expression of pro-inflammatory cytokines, specifically IL-6 and IL-1β, during the critical inflammation resolution phase in innate myeloid cells and macrophages, respectively (13). Deficiency of TET2 leads to upregulated IL-1β expression and unexpected cleavage of IL-1β mediated by the NOD-like receptor protein 3 (NLRP3) inflammasome, unequivocally underscoring TET2’s indispensable role in promoting inflammation resolution (117). **Figure 7** graphically summarizes the role of TET2 in CVD.

Clonal hematopoiesis (CH) originates from somatic mutations that confer a selective fitness advantage to hematopoietic stem cells, leading to the expansion of blood cell clones. CH commonly involves gene variants that mediate epigenetic modifications *(TET2, DNMT3A, ASXL1)* or cytokine signaling *(JAK2)*, CH has emerged as an independent risk factor for CVD, a process underpinned by macrophage inflammasome activation (118).

TET2 deficiency drives hypermethylation within the promoter of dual specificity phosphatase 10 *(DUSP10)*. As DUSP10 specifically dephosphorylates c-Jun N-terminal kinase 1 (JNK1), its loss results in exacerbated JNK1 phosphorylation (at Thr183/Tyr185) and subsequent activation of downstream signaling (119, 120). This heightened JNK1 signaling, in turn, promotes the deubiquitination and subsequent activation of the NLRP3 inflammasome by the deubiquitinating enzyme BRCA1/BRCA2-containing complex subunit 3 (BRCC3) (120). Murine models of TET2 CH-driven accelerated atherosclerosis and neutrophil extracellular trap formation (NETosis) demonstrate reversibility upon treatment with geraldol, a BRCC3 deubiquitinase inhibitor, or through hematopoietic depletion of abraxas brother 1 (Abro1), a critical scaffold protein of the BRCC3-containing cytoplasmic complex (120, 121).

Another study indicates that vascular smooth muscle cells (VSMCs) possess remarkable plasticity. This plasticity refers to their ability to reversibly switch between a contractile phenotype (differentiated state) and a dedifferentiated phenotype (proliferative, migratory state) in response to different environmental stimuli (122). Their reversible differentiation is necessary for growth and wound healing but can also contribute to pathologies including atherosclerosis and restenosis (123). Serum response factor (SRF), a widely expressed transcription factor, forms a stable complex with myocardin (MYOCD), a smooth muscle-specific coactivator, in VSMCs (MYOCD/SRF) (122, 123). This complex, along with Krüppel-like factor 4 (KLF4), has been identified as a key transcriptional regulator driving VSMC phenotypic plasticity, promoting the contractile and dedifferentiated phenotypes (124). TET2 acts as a pivotal epigenetic regulator of VSMC differentiation and the phenotypic switch towards a pro-proliferative and migratory state, acting upstream of MYOCD/SRF and KLF4 (122, 125). By promoting the expression of crucial contractile genes, TET2 induces a differentiated, contractile smooth muscle cell (SMC) phenotype, thereby ameliorating vascular injury in human atherosclerotic disease (122).

Moreover, coiled-coil domain containing 80 (CCDC80) inhibits the phosphorylation of ERK1/2, concurrently reducing TET2 expression (126). This reduction consequently elevates the methylation level in the lipoprotein lipase *(LPL)* promoter region, and impairs the interaction between TET2 and the transcription factor forkhead box O3a (FOXO3a) (127). These events collectively lead to reduced LPL expression and ultimately accelerate atherosclerosis (126).

Oxidized low-density lipoprotein (oxLDL) is a pro-atherogenic factor. Research has revealed that miR-125a-5p targets the 3’-UTR of *TET2* mRNA, suppressing *TET2* expression during immune responses, leading to oxLDL-induced pyroptosis in VECs (128). Inhibition of TET2 activates NF-κB, which in turn upregulates the expression and activity of the NLRP3 inflammasome and caspase-1, an inflammatory cysteine aspartase, culminating in VEC pyroptosis (128). As a DNA demethylase, downregulation of TET2 leads to aberrant DNA methylation. This epigenetic perturbation contributes to mitochondrial dysfunction, increasing the production of reactive oxygen species (ROS) (128, 129). Excessive ROS acts as a “danger signal” to activate NLRP3, also ultimately leading to caspase-1 activation (128, 130). Activated caspase-1 triggers cell membrane pore formation, DNA fragmentation, and the release of mature IL-1β and IL-18, causing a sterile inflammatory response (131). This further leads to pyroptotic cell death, subsequently promoting atherosclerosis (128).

## 6 The role of TET2 in metabolic diseases

While TET2’s roles in cancer and inflammation are well-documented, its epigenetic regulatory functions, primarily through DNA demethylation, are also increasingly recognized as pivotal in metabolic physiology. Accumulating evidence highlights TET2’s critical involvement in maintaining metabolic homeostasis and its dysregulation in various metabolic disorders.

### 6.1 Diabetes

Type 1 diabetes (T1D) is driven by autoimmune injury of pancreatic β-cells. Acute and chronic infections boost pancreatic IFN-α, which upregulates polyribonucleotide nucleotidyltransferase 1 *(PNPT1)*, to degrade miR-26a, relieving the post-transcriptional repression of *TET2*. This leads to *TET2* overexpression and elevated global 5hmC modification in β-cells, ultimately triggering islet autoimmunity (132). Further animal and human islet experiments confirm that inflammatory cytokines including IFNγ, IL-1β and TNF-α also upregulate TET2 (133). Accumulated TET2 enriches 5hmC at the promoters of inflammatory genes, unlocks binding sites for STAT and IRF transcription factors, stimulates chemokine secretion, and recruits pathogenic Tregs to infiltrate pancreatic islets (133). In brief, diverse inflammatory stimuli induce TET2 overexpression via miRNA regulation, and the resultant widespread 5hmC deposition amplifies inflammatory cascades to facilitate the progression of type 1 diabetes.

Relevant studies have demonstrated that hepatocyte nuclear factor 4α (HNF4α) recruits TET2 to the promoter region of fructose-1,6-bisphosphatase (*FBP1*), the rate-limiting enzyme for gluconeogenesis (134). TET2 mediates demethylation to activate *FBP1* transcription and regulate hepatic glucose output (134). Metformin induces the phosphorylation of HNF4α, which impedes the recruitment of TET2 to the *FBP1* promoter by HNF4α, thereby repressing FBP1 expression, reducing glucose production, and alleviating pathological manifestations of type 2 diabetes (T2D) (134).

**Figure 8:**
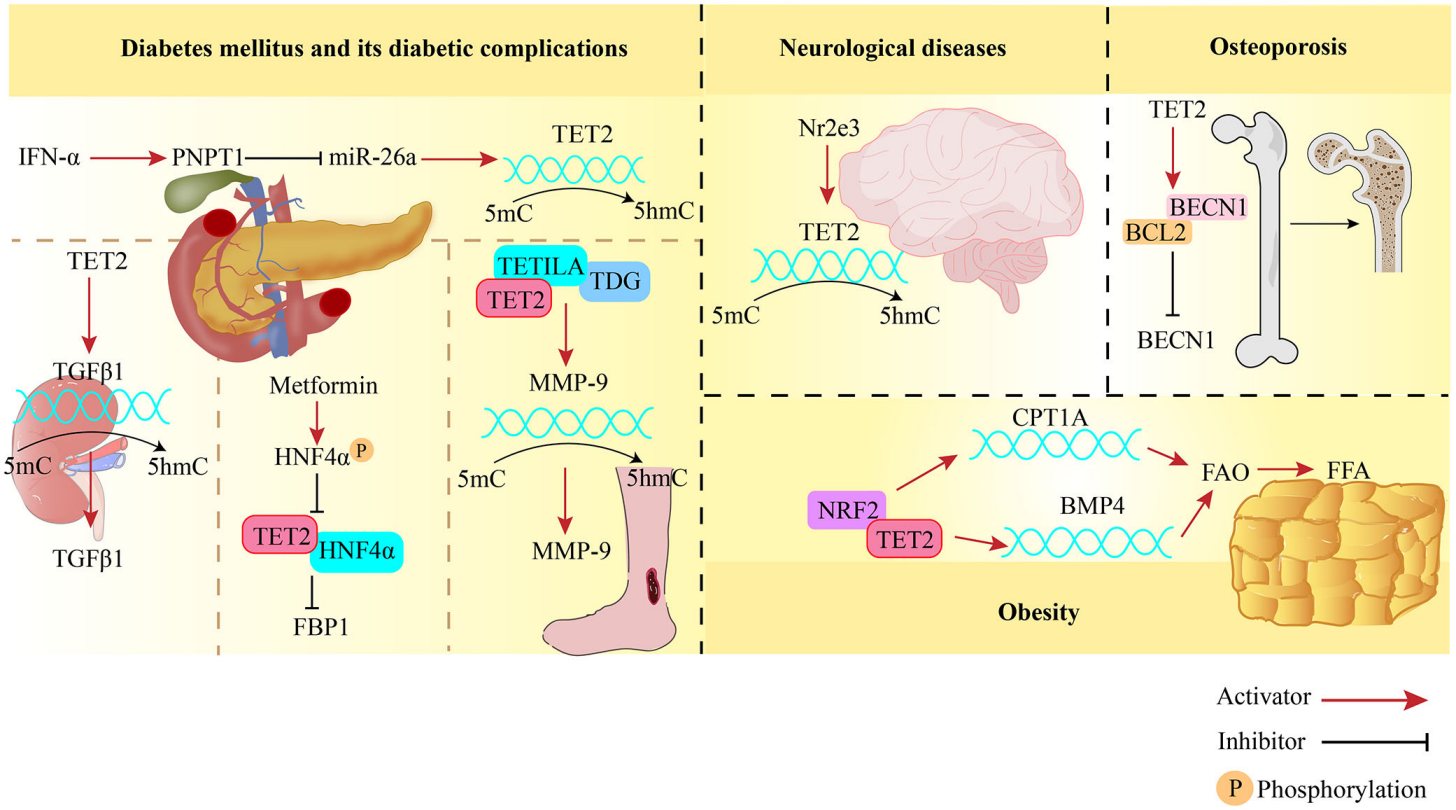
In T1D, IFN-α mediates crosstalk between pancreatic β-cells and immunity to drive autoimmunity. Upon infection, IFN-α upregulates PNPT1, degrading miR-26a via disrupted 3’-UTR regulation and boosting TET2 levels. In addition, metformin promotes the phosphorylation of HNF4α, impairs the capacity of HNF4α to recruit TET2, blocks the binding of TET2 to the *FBP1* promoter, and ultimately suppresses the expression of FBP1. In diabetic nephropathy, TET2 activates TGFβ1 expression through CpG island demethylation. Additionally, the TETILA recruits TET2 and TDG to the *MMP-9* promoter, enhancing *MMP-9* expression and contributing to delayed wound healing in diabetes. In adipose tissue, TET2 cooperates with NRF2 to modulate the methylation of *CPT1A* and *BMP4*, promoting WAT browning and FAO, counteracting obesity. In bones, TET2 regulates osteoclast differentiation through autophagy modulation by inhibiting BCL2 and facilitating BECN1 activation, which contributing to postmenopausal osteoporosis-related bone loss. In the brain, *NR2E3* enhances *TET2* expression by binding its promoter, influencing neuronal differentiation and depressive behavior, particularly under chronic stress conditions. In the figure, arrows denote promotion, and T-shaped symbols denote inhibition.

Further research indicates that TET2 activates transforming growth factor beta 1 (TGFβ1) expression through the demethylation of CpG islands in the regulatory region of *TGFβ1*, playing a significant role in the pathogenesis of diabetic nephropathy (DN) (135). Additionally, the TET2-interacting long noncoding RNA (TETILA) recruits TET2 and TDG to form a demethylation complex at the matrix metalloproteinase-9 *(MMP-9)* promoter (**Figure 8**) (136). This interaction consequently enhances MMP-9 expression, a factor strongly implicated in delayed diabetic wound healing.

### 6.2 Obesity

Obesity is a complex metabolic disorder characterized by excessive accumulation of body fat (137). Research has found that endothelial TET2 regulates HFD-induced obesity by modulating white adipose browning and adipocyte lipolysis (137). Nuclear factor erythroid-2-related factor 2 (NRF2) is crucial for the adaptive browning of white adipocytes (137, 138). Endothelial TET2 interacts with NRF2 to regulate white adipose tissue (WAT) browning, promoting fatty acid oxidation (FAO) and lipolysis in adipocytes (138). This NRF2-TET2 complex modulates the methylation status of the carnitine palmitoyl transferase 1A *(CPT1A)* and bone morphogenetic protein 4 *(BMP4)* promoters. Specifically, the NRF2-TET2 complex regulates the expression level of CPT1A, directly modulating FAO (137). Furthermore, it modulates BMP4 secretion, which in turn affects the production of free fatty acids (FFAs), a key FAO substrate (**Figure 8**).

### 6.3 Osteoporosis

Increased bone resorption by osteoclasts following estrogen deficiency is the primary cause of postmenopausal osteoporosis (139). Macroautophagy, commonly referred to as autophagy, is a fundamental cellular process that maintains cellular homeostasis by recycling unnecessary and damaged organelles (140). Studies indicate that TET2 exacerbates bone loss in ovariectomized (OVX) mice and promotes osteoclast differentiation by regulating autophagy (139). Under conditions mimicking postmenopausal estrogen deficiency, TET2 expression is upregulated, which suppresses the expression of B cell lymphoma 2 (BCL2). This suppression liberates beclin 1 (BECN1) from inhibitory binding to BCL2, thereby activating autophagy (141). This autophagy induction ultimately enhances osteoclast differentiation and contributes to bone loss. Critically, genetic knockdown of *TET2* can attenuate bone loss by counteracting these mechanistic events (**Figure 8**).

## 7 The role of TET2 in neurological diseases

As a DNA hydroxymethylase, TET2 is implicated in the pathogenesis of various neurological disorders, including Alzheimer’s disease (AD), Parkinson’s disease (PD) and multiple sclerosis (142). In AD, TET2 dysfunction is closely associated with key pathological processes such as amyloid-β (Aβ) deposition and Tau hyperphosphorylation (143–145). Studies demonstrate widespread disruption of genomic 5hmC landscapes in both AD models and patient brains, particularly within gene regions governing synaptic plasticity and neuronal survival (146), suggesting that TET2-mediated epigenetic dysregulation may be a critical mechanism underlying cognitive decline in AD. TET2 contributes to neuronal differentiation by modulating 5hmC levels at neuronal enhancers. *In vivo* inactivation of TET2 protects dopaminergic neurons from inflammation-induced neurotoxicity (147), attenuates the transcriptional inflammatory response and suppresses microglial activation. TET2 upregulation significantly influences brain inflammatory responses by affecting early transcription of immune-related genes and later-phase inflammatory activity (148). However, the current understanding of TET2’s precise roles in PD and multiple sclerosis remains nascent, warranting more extensive and focused investigation.

The role of TET2 in neurodegenerative diseases exhibits significant cell-type and disease-specificity. In AD, TET2 deficiency exacerbates neuroinflammation and cognitive decline (148, 149). Notably, in a mouse model of Parkinson’s disease (PD), TET2 deficiency appeared neuroprotective, highlighting the need for disease-specific considerations in TET2-targeted therapeutic strategies (147).

Major depressive disorder (MDD) is a complex neuropsychiatric condition, characterized by persistent low mood, anhedonia, apathy, and, in severe cases, suicidal ideation. Epigenetic modifications represent the product of gene-environment crosstalk, and epigenetic mechanisms are key contributors to both MDD and chronic stress exposure (150). Specifically, dysregulation of DNA methylation (5mC) and hydroxymethylation (5hmC) patterns is strongly associated with the development of depressive states. Research highlights nuclear receptor subfamily 2 group E member 3 (NR2E3) as a crucial transcription factor for *TET2*. NR2E3 directly binds to the promoter region of *TET2*, increasing its transcriptional activity and subsequent expression (151). Murine studies indicate that hippocampal knockdown of *NR2E3* results in diminished *TET2* expression and the manifestation of depressive-like behaviors (151). Mechanistically, NR2E3 collaborates with the RNA helicase DExH-box helicase 30 (DHX30) to enhance *TET2* transcription (151). Under chronic stress conditions, the expression of *NR2E3* in the hippocampal region is upregulated, concurrently inhibiting the activity of the histone demethylase lysine-specific demethylase 1 (LSD1), which further promotes the generation of *TET2* mRNA (**Figure 8**) (151).

## 8 Drugs associated with TET2 regulation

### 8.1 Mutant *IDH1/2* inhibitors

Somatic mutations frequently occur within the conserved active sites of IDH1/2 across various tumors. In gliomas and leukemias, these mutations cause single amino acid substitutions, typically at arginine 132 (R132) in IDH1 or the corresponding arginine 172 (R172) in IDH2, or at arginine 140 (R140) in IDH2 in leukemias (152–154). Mutant *IDH1* and *IDH2* reduce α-KG to the oncometabolite R-2-hydroxyglutarate (R-2-HG) (153, 154). 2-HG acts as a competitive inhibitor for various α-KG-dependent dioxygenases, including the TET family (155). This competitive inhibition diminishes 5hmC generation, leading to aberrant DNA methylation patterns and compromised hematopoietic differentiation. Consequently, mutant *IDH1/2* inhibitors can lower intracellular 2-HG levels, thereby restoring TET2 function and promoting cellular differentiation (156).

### 8.2 Exportin 1 inhibitors

XPO1, is a nuclear export receptor critical for cellular homeostasis, mediating the translocation of diverse cargoes, including proteins and various RNA species, from the nucleus to the cytoplasm (157, 158). Dysregulation of XPO1 is implicated in the pathogenesis of various solid tumors and hematological malignancies (159, 160). Studies utilizing *TET2*-mutant zebrafish models have shown that XPO1 inhibitors exhibit selective cytotoxicity towards *TET2*-mutant HSPCs, ultimately inducing apoptosis in these malignant cells (161).

### 8.3 Topoisomerase 1-targeting drugs and PARP1 inhibitors

Research indicates that TOP1-targeting drugs and PARP1 inhibitors can selectively kill *TET2*-mutant HSPCs. *TET2*-mutant cells exhibit reduced expression of tyrosyl-DNA phosphodiesterase 1 (TDP1). TOP1 inhibitors cause the accumulation of Top1-linked DNA covalent cleavage complexes (TOP1cc), leading to lethal DSBs (162, 163). Concurrently, PARP1 inhibitors exacerbate single-strand break (SSB) repair deficiencies, thereby creating a “repair dilemma” that synthetically is lethal to the mutant cells (162, 163). This synthetic lethality approach offers a promising therapeutic strategy for *TET2* mutation-associated hematologic malignancies, enabling specific targeting of malignant cells with minimal adverse effects on healthy tissues by employing low doses of TOP1 or PARP1 inhibitors.

### 8.4 Dietary valine

HDAC6, a human deacetylase, directly binds valine via its unique SE14 domain. Intracellular valine deficiency disrupts this binding, triggering HDAC6 translocation into the nucleus (164). Within the nucleus, the enzymatically active region of HDAC6 binds to the catalytic domain of the DNA hydroxymethylase TET2 (164). This interaction promotes TET2 deacetylation, thereby activating its enzymatic activity (164). This cascade subsequently initiates DNA demethylation and induces DNA damage, ultimately contributing to tumor growth inhibition (164).

### 8.5 Canakinumab

Canakinumab is a fully human neutralizing monoclonal antibody that specifically binds to IL-1β and blocks its downstream inflammatory cascade (165). Clonal hematopoiesis of indeterminate potential (CHIP) carrying *TET2* mutations activates the NLRP3 inflammasome, driving the production of large amounts of mature IL-1β and exacerbating systemic chronic inflammation. Intervention with canakinumab can inhibit the pro-inflammatory signaling downstream of IL-1β, thereby reducing the risk of atherosclerotic adverse cardiovascular events and solid malignant tumors.

### 8.6 Vitamin C

As a cofactor and agonist of TET2, Vitamin C exerts broad epigenetic regulatory effects by activating TET2 (166). In RCC, Vitamin C maintains the demethylated state of the *IRF1* promoter and upregulates IRF1 expression through the IFN-γ-STAT1 signaling pathway (67). Vitamin C enhances TET2 activity, promotes demethylation at the CNS2 region of the *FOXP3* gene, and thereby stabilizes Treg function in patients with AR (107). High-dose Vitamin C restores TET2 protein function, suppresses aberrant proliferation of hematopoietic stem cells and retards the progression of myeloid malignancies (167, 168). Dietary vitamin supplementation maintains the physiological homeostasis of hematopoietic stem cells and impedes malignant transformation in the setting of clonal hematopoiesis (168). In summary, by enhancing the catalytic activity of TET2, Vitamin C epigenetically regulates core signaling and immune-associated genes. It alleviates AR and exerts anti-tumor inhibitory effects against RCC and myeloid malignancies.

## 9 Conclusions and future perspectives

This review systematically summarizes the structural features, catalytic activity and non-catalytic functions of TET2, a core epigenetic regulator, elucidates its multi-layer regulatory mechanisms in tumors, inflammatory diseases, cardiovascular disorders, metabolic illnesses and neurological diseases, consolidates cutting-edge research advances in TET2-targeted interventions, and establishes a cross-disease regulatory network centered on TET2. As an α-KG and Fe^2+^-dependent dioxygenase, *TET2* maintains genomic epigenetic homeostasis via the canonical DNA demethylation pathway (3). It catalyzes the stepwise oxidation of 5mC into 5hmC, 5fC and 5caC, followed by the initiation of the base excision repair cycle (2, 3). Beyond mediating DNA epigenetic modification, TET2 oxidizes RNA m^5^C to participate in post-transcriptional regulation. It can also engage in biological processes such as inflammation resolution and chromatin remodeling through protein-protein interactions independent of its enzymatic activity. Therefore, TET2 acts as a central hub linking epigenetic modifications, cellular homeostasis and the pathogenesis of various diseases.

Diseases regulated by TET2 constitute intricate regulatory networks characterized by crosstalk between diverse cell types and tissues, which endows TET2 with complicated, even opposing regulatory roles throughout disease initiation and progression. Hematopoietic stem cells, myeloid progenitors, and diverse immune cells in the tumor microenvironment originate from distinct cell lineages and exhibit divergent epigenetic responses following TET2 deficiency (44–46, 104). Signaling crosstalk between tumor cells and immune cells further amplifies the dual regulatory properties of TET2 (13, 50, 56, 62, 65, 67, 116). Accordingly, most existing relevant studies rely heavily on single-cell sequencing to resolve cellular heterogeneity. Conventional bulk sequencing masks molecular differences among cell populations and thus tends to generate one-sided and conflicting experimental results. This also indicates that subsequent TET2-related research must fully take into account cell subtypes, genetic backgrounds, temporal and spatial dynamic changes, as well as discrepancies arising from different experimental models ranging from cell lines, animal models to human tissues.

Repurposing existing drugs by targeting the TET2 pathway has become a vital research direction in translational medicine, with metformin and Vitamin C serving as typical representatives highlighted in this review. Metformin suppresses hepatic gluconeogenesis through the HNF4α-TET2 axis to ameliorate type 2 diabetes, and concurrently activates AMPK signaling to upregulate TET2 expression, thereby exerting dual effects of anti-tumor activity and glucose metabolism regulation (50, 134). As an agonist of the TET2, Vitamin C mediates distinct epigenetic regulatory functions in RCC, AR and HCC, producing biological effects including anti-tumor activity and stabilization of Tregs (67, 69, 107). Both drugs have been widely applied in clinical practice with comprehensive safety data, conferring promising translational potential.

In conclusion, TET2 participates in the initiation and progression of systemic multi-organ diseases via multiple epigenetic regulatory pathways, and its functional activity is co-governed by cell types, disease stages and experimental models. Classic pharmaceuticals such as metformin and Vitamin C can activate the TET2 pathway in a targeted manner, providing simple and feasible translational strategies for epigenetic intervention against diverse diseases, and laying a theoretical foundation for the subsequent development of highly specific TET2-targeted novel drugs.

## Glossary

TET: Ten-eleven translocation
α-KG: α-ketoglutarate
5mC: 5-methylcytosine
5hmC: 5-hydroxymethylcytosine
5fC: 5-formylcytosine
5caC: 5-carboxylcytosine
TDG: Thymine DNA glycosylase
m^5^C: 5-methylcytosine
hm^5^C: 5-hydroxymethylcytosine
HSC: Hematopoietic stem cell
CVD: Cardiovascular diseases
HDAC: Histone deacetylase
IL: Interleukin
DSβH: Double-stranded β-helix
CpG: Cytosine-phosphate-guanine
AML: Acute myeloid leukemia
MDS: Myelodysplastic syndromes
MPN: Myeloproliferative neoplasms
HSPCs: Hematopoietic stem and progenitor cells
ESC: Embryonic stem cell
MBD6: Methyl-CpG binding domain protein 6
caRNAs: Chromatin-associated RNAs
H2AK119ub: Monoubiquitinated Lys119 of histone H2A
LSCs: Leukemia stem cells
TSPAN13: Tetraspanin 13
CXCR: C-X-C chemokine receptor type
CXCL: C-X-C motif chemokine ligand
BM: Bone marrow
YBX1: Y-box binding protein 1
SOX2: Sex-determining region Y-box 2
STAT: Signal transducer and activator of transcription
IFN: Interferon
IRF1: Interferon regulatory factor 1
PD-L1: Programmed death-ligand 1
KMT2D: Histone-lysine N-methyltransferase 2D
ARID1A: AT-rich interaction domain 1A
BCLAF1: B-cell lymphoma-2-associated transcription factor 1
ASCL2: Achaete-scute homolog 2
AMPK: AMP-activated protein kinase
EC: Endometrial cancer
Ser99: Serine 99
AST: Adeno-to-squamous transition
GBM: Glioblastoma
lncRNAs: Long non-coding RNAs
CD: Catalytic domain
ALI: Acute lung injury
ITA: Itaconate
ITGA10: Integrin α10
PI3K: Phosphatidylinositol 3-kinase
COPD: Chronic obstructive pulmonary disease
AR: Allergic rhinitis
GPX4: Glutathione peroxidase 4
NF-κB: Nuclear factor κB
AKI: Acute kidney injury
PPAR: Peroxisome proliferator-activated receptor
ARDS: Acute respiratory distress syndrome
LPS: Lipopolysaccharide
CS: Cigarette smoke
CSE: Cigarette smoke extract
FOXP3: Forkhead box P3
TSDR: Treg-specific demethylated region
HI: Hypoxic-ischemic
NLRP3: NOD-like receptor protein 3
SMC: Smooth muscle cell
oxLDL: Oxidized low-density lipoprotein
DUSP10: Dual specificity phosphatase 10
JNK1: c-Jun N-terminal kinase 1
BRCC3: BRCA1/BRCA2-containing complex 3
MYOCD: Myocardin
SRF: Serum response factor
Abro1: Abraxas brother 1
KLF4: Krüppel-like factor 4
CCDC80: Coiled-coil domain containing 80
FOXO3a: Forkhead box O3a
LPL: Lipoprotein lipase
ROS: Reactive oxygen species
CH: Clonal hematopoiesis
VSMCs: Vascular smooth muscle cells
T1D: Type 1 diabetes
PNPT1: Polyribonucleotide nucleotidyltransferase 1
TGFβ1: Transforming growth factor beta 1
MMP-9: Matrix metalloproteinase-9
NRF2: Nuclear factor erythroid-2-related factor 2
CPT1A: Carnitine palmitoyl transferase 1A
BMP4: Bone morphogenetic protein 4
FAO: Fatty acid oxidation
BCL2: B cell lymphoma 2
BECN1: Beclin 1
TETILA: TET2-interacting long noncoding RNA
AD: Alzheimer’s disease
PD: Parkinson’s disease
MDD: Major depressive disorder
NR2E3: Nuclear receptor subfamily 2 group E member 3
IDH: Isocitrate dehydrogenase
2-HG: 2-hydroxyglutarate
XPO1: Exportin 1
Top1: Topoisomerase 1
MIP: Macrophage inflammatory protein.
